# Standard Automated Perimetry versus Matrix Frequency Doubling Technology Perimetry in Subjects with Ocular Hypertension and Healthy Control Subjects

**DOI:** 10.1371/journal.pone.0057663

**Published:** 2013-02-28

**Authors:** Julia Lamparter, Shakhsanam Aliyeva, Andreas Schulze, Manfred Berres, Norbert Pfeiffer, Esther M. Hoffmann

**Affiliations:** 1 Department of Ophthalmology, University Medical Centre Mainz, Mainz, Germany; 2 Institute of Medical Biometry, Epidemiology and Informatics, University Medical Centre Mainz, Mainz, Germany; 3 University of Applied Sciences Koblenz, RheinAhrCampus Remagen, Remagen, Germany; Eye Hospital, Charité, Germany

## Abstract

**Background:**

To evaluate the relationship and agreement between standard automated perimetry (SAP) and Matrix frequency doubling technology (Matrix-FDT) in subjects with ocular hypertension and healthy control subjects.

**Methods:**

Forty-four eyes of 44 ocular hypertensive subjects and 29 eyes of 29 healthy age-matched control subjects were included in this prospective study. All participants underwent complete ophthalmic examination, including slit-lamp biomicroscopy, intraocular pressure measurement, pachymetry, and dilated fundus examination, and showed reliable visual field tests. One randomly selected eye of each participant was examined with SAP (Swedish Interactive Threshold Algorithm [SITA] Standard 24-2 test) and Matrix-FDT (24-2 threshold test), in random order. Correlations between global indices (MD, PSD), regions (2 hemifields, 4 quadrants, 6 sectors) and 52 single field positions were analyzed using Spearman’s rank correlation coefficient.

**Results:**

In both groups, mean deviation values of SAP and Matrix-FDT correlated significantly (OHT subjects: r = 0.47, p<0.005; healthy subjects: r = 0.68; p<0.001, respectively). Pattern standard deviation of SAP and Matrix-FDT showed no significant correlation in healthy subjects but correlated significantly in ocular hypertensive subjects (r = 0.45, p<0.005). In healthy subjects, a significant correlation between SAP and Matrix-FDT was shown in the supero-temporal and infero-temporal sectors of the disc (r = 0.40 and r = 0.38, p<0.05, respectively). In OHT subjects, supero-temporal, supero-nasal and nasal sectors correlated significantly (r = 0.49, 0.62 and 0.38, p≤0.01, respectively). The correlation pattern of individual visual field test locations appeared heterogeneous in both groups.

**Conclusions:**

In both, ocular hypertensive and healthy subjects SAP and Matrix-FDT correlate well. In ocular hypertensive subjects, both techniques showed good correlation in the supero-temporal, supero-nasal, and nasal sectors of the disc. Poor agreement was found in the temporal, infero-temporal and infero-nasal disc sectors. This missing correlation might be related to early retinal nerve fiber layer damage in these regions of the disc, recognized by one of the visual field instruments.

## Introduction

Glaucoma is a disease in which death of retinal ganglion cells (RGCs) is associated with visual field defects and visual impairment. The evaluation of these visual field defects is essential for diagnosing and monitoring glaucoma and for recognizing progression. Until today the “gold standard” for visual field testing is standard automated perimetry (SAP) which measures light sensitivity thresholds at various locations across the visual field. [1], [2] However, up to a high percentage of retinal ganglion cells (RGC) must be lost before a scotoma is detected with SAP. [3], [4] Investigators have been interested in finding diagnostic techniques that would allow earlier detection of visual field loss than those detected by standard white-on-white perimetry. [5].

Frequency doubling technology (FDT) has been suggested a test that may detect retinal ganglion cell damage earlier than standard automated perimetry. In comparison to SAP, FDT uses contrast sensitivity and a different test algorithm to detect visual field changes. The second generation of FDT perimeter, the Humphrey Matrix-FDT, was released in 2003. It utilizes additional tests to improve the spatial resolution of visual field defects by using smaller targets with a higher spatial frequency and a lower temporal frequency. [6], [7] Comparison with standard FDT perimetry suggests that Matrix perimetry may have higher sensitivity for early glaucomatous loss and better characterization of the pattern of visual field loss. [8] Therefore, Matrix perimetry may provide additional benefits for monitoring subtle progression in glaucomatous field defects. [9] Several investigations have affirmed the first and second generation FDT excellent results in detecting early, moderate and advanced visual field loss in patients with glaucoma [2], [4], [5], [7], [8], [10], [11], [12], [13], [14], [15] even though there has been described a tendency to miss some early defects. [1], [7], [16] Additional investigations have shown that FDT may have preferable variability characteristics to SAP [16], [17] because it exhibits significantly less within- and between-test variability at both, normal and reduced sensitivity levels. [18] Whereas a large number of studies have been published in the past comparing the first-generation FDT with standard automated perimetry, fewer studies have compared the second generation Matrix-FDT with SAP. The majority of these studies included glaucoma patients, [1], [2], [6], [7], [10], [19], [20] whereas only few studies included ocular hypertensive subjects. [9], [13] Persons with ocular hypertension have an increased risk of developing glaucoma during their lifetime. They need to be followed-up regularly in order to recognize conversion to glaucoma. It is important to find techniques that are able to detect glaucomatous damage earlier than SAP in order to avoid any significant nerve fiber loss in OHT subjects.

The purpose of this cross sectional observational study was to evaluate the agreement between Matrix frequency doubling technology perimetry and standard automated perimetry (SITA 24-2 standard algorithm) in subjects with ocular hypertension and healthy subjects and to determine if Matrix-FDT might be a helpful tool in detecting early visual field defects in OHT subjects that are not yet detectable with standard white-on-white perimetry.

## Methods

Forty-four eyes of 44 ocular hypertensive subjects and 29 eyes of 29 healthy control subjects were included in this prospective controlled study.

The study protocol was approved by the Ethics Committee of Mainz University, Rhineland Palatinate, Germany. All study procedures adhered to the recommendations of the Declaration of Helsinki and all participants signed informed consent prior to any testing, clinical examination, or collection of demographic or medical information.

Ocular hypertension was defined as IOP>21 mmHg on at least two occasions prior to enrolment. At the time of enrolment, subjects with OHT were allowed to be on IOP lowering treatment. All participants had normal results on Humphrey 24-2 automated perimetry tests (no clustered field defects) as well as healthy optic nerves by clinical expert exam (dilated stereoscopic fundus examination with a high power fundus lens of 78 D or 90 D). Discs were considered healthy when they had symmetric vertical cup to disc ratio in both eyes, intact neuroretinal rims without notching, no disc hemorrhages, and no nerve fiber layer defect. Age-matched control subjects had an intraocular pressure <21 mmHg, and normal disc appearance based on a dilated stereoscopic fundus examination. All measurements were performed on one day, and all participants were recruited within an eight-month period. All subjects were familiar with standard automated perimetry because each had undergone at least one visual field examination before the study. None of the participants had undergone frequency doubling perimetry testing prior to the study. A brief demonstration was performed to familiarize them with the procedure. Subjects who met the inclusion criteria and who agreed to participate were enrolled in the study.

### Participants

#### Inclusion criteria

To be included in the study, participants had to have best-corrected visual acuity of at least logMAR 0.3, spherical refraction within ±5.0 D, and astigmatism of less than ±3.0 D. All subjects underwent complete ophthalmic examination, including visual acuity testing, slit-lamp biomicroscopy, intraocular pressure measurement with Goldmann applanation tonometry, central corneal thickness measurement with ultrasound pachymetry, and dilated fundus examination.

#### Exclusion criteria

Subjects with non-glaucomatous secondary causes of elevated intraocular pressure (e.g., iridocyclitis, trauma), other intraocular disease, history of other conditions that might affect visual field testing (e.g.pituitary lesions, demyelinating diseases, diabetic retinopathy), or medications known to affect visual field sensitivity, were excluded from the investigation.

### Procedures

#### Visual field testing in this study

Testing on all subjects was performed using the Matrix Frequency Doubling Technology perimeter (Humphrey Matrix, Carl Zeiss Meditec, Jena, Germany) and the Humphrey Field Analyzer II (Carl Zeiss Meditec, Jena, Germany) in randomized order on one day. If required, a rest of 15 minutes between each visual field exam was permitted. For both, OHT and healthy subjects, one eye was randomly selected to be included in the study, and the investigator was masked to the subject’s group classification.

Participants were tested on the Humphrey Field Analyzer II, using the 24-2 standard test algorithm (Swedish Interactive Threshold Algorithm; SITA) which consists of 52 test locations. On FDT, the 24-2 full threshold test algorithm, consisting of 55 test locations, was used. The ZEST (zippy estimation of sequential testing) algorithm was used for threshold estimation. Not included in the comparison were the two locations in the vicinity of the blind spot and the test point in the central visual field which are examined only by the FDT but not by the SAP perimeter.

### Data Analysis

The statistical analysis evaluated the agreement between Humphrey-SAP and Matrix-FDT in subjects with OHT and healthy controls.

Comparisons between global indices (Humphrey MD/PSD versus Matrix-FDT MD/PSD) and between groups were based on t-tests. Correlations between global indices, hemifields, quadrants, sectors and single field points were compared using Spearman’s rank correlation coefficient (r). The significance level was set to p<0.05.

This study is an explorative study where no p value adjustment was performed. This strategy was chosen since adjustment for multiple testing (in quadrants, hemifields, sectors and single field points) would have increased the risk of falsely accepting the null hypothesis (type II error). Especially Bonferroni adjustments are known to be highly conservative so that one can miss real differences. [21], [22], [23] Instead, the number of observed significant correlations was compared with the number of expected significant correlations in order to get an impression if the findings make sense above chance level.

## Results

Forty-four eyes of 44 ocular hypertensive subjects and 29 eyes of 29 healthy control subjects were included in this prospective controlled study. Table 1 gives an overview of all demographic data and clinical characteristics of both study groups.

**Table 1:** Demographic information and clinical characteristics of all participants.

	OHT group (n = 44)	control subjects (n = 29)	p-value
**Mean age (yrs) ± SD (range)**	61.1±9.5 (34–76)	59.8±8.5 (47–71)	0.54
**Male**	16	8	
**Right eyes**	23	16	
**Mean visual acuity (logMAR) ± SD (range)**	0.04±0.07 (0.0–0.3)	0.01±0.04 (−0.1–0.1)	<0.01
**Mean IOP (mmHg) ± SD (range)**	20.9±3.5 (12.0–27.0)	14.1±2.9 (8.0–20.0)	<0.001
**Mean central corneal thickness (µm) ± SD (range)**	578±42 (496–664)	548±37 (490–631)	<0.05
**Mean FDT test duration (sec.) ± SD (range)**	311±14.0 (292–356)	305±8.7 (294–328)	0.05
**Mean SAP test duration (sec.) ± SD (range)**	312±38.2 (256–401)	303±36.4 (254–420)	0.31

Abbreviations: OHT = ocular hypertension; FDT = frequency doubling technology; SAP = standard automated perimetry; yrs = years; SD = standard deviation; IOP = intraocular pressure; sec. = seconds.

Among the 44 OHT subjects, SAP glaucoma hemifield test result was classified as “inside normal limits” in 32 eyes, “outside normal limits” in 10 eyes, and “others” in 2 eyes (including borderline). The FDT glaucoma hemifield result was classified as “normal” in 28 eyes, “outside normal limits” in 7 eyes, and “others” in 9 eyes (including borderline). Complete concordance between SAP and FDT glaucoma hemifield test was shown in 28 (64%) eyes (24 times “inside normal limits”, 4 times “outside normal limits”). SAP glaucoma hemifield test was normal in 8 cases in which FDT showed different results (6 times “borderline” and 2 times “outside normal limits”).

Among the 29 healthy subjects, the SAP glaucoma hemifield test result was classified as “inside normal limits” in 22 eyes, “outside normal limits” in 3 eyes, and “others” in 4 eyes (including borderline). The FDT glaucoma hemifield result was classified as “normal” in 22 eyes, “outside normal limits” in 4 eyes, and “others” in 3 eyes (including one eye with abnormal high sensitivity and 2 eyes with borderline test result). Complete concordance of GHT between SAP and FDT was shown in 19 (66%) eyes (17 times “inside normal limits”, 1 time “outside normal limits”, 1 time “borderline”). SAP glaucoma hemifield test was normal in 4 cases in which FDT showed different results (3 times “outside normal limits” and 1 time “borderline”).

Table 2 shows the comparison between global indices of SAP and Matrix-FDT in both groups. Global indices for SAP and Matrix-FDT (MD and PSD) did not differ statistically significant between ocular hypertensive subjects and healthy control subjects. However, significances became statistically significant when comparing SAP MD versus FDT MD and SAP PSD versus FDT PSD within both groups.

**Table 2:** Comparison between global indices of SAP and Matrix-FDT in ocular hypertensive subjects and healthy control subjects (MD: mean deviation, PSD: pattern standard deviation).

	OHT group	Control subjects	Comparison of mean values of OHT versus control subjects (p-value)
**SAP MD (dB) ± SD (range)**	−0.48±1.40 (−4.24–1.75)	−0.22±1.10 (−2.25–2.12)	0.41
**SAP PSD (dB) ± SD (range)**	1.95±0.95 (0.94–5.30)	1.63±0.40 (1.16–2.99)	0.10
**FDT MD ± SD (range)**	0.25±2.52 (−5.31–4.63)	0.71±2.51(−6.21–5.81)	0.45
**FDT PSD ± SD (range)**	2.83±0.73 (2.09–5.53)	2.62±0.37 (1.92–3.31)	0.16
**Comparison of SAP MD versus** **FDT MD (p-value)**	**0.03**	**0.02**	
**Comparison of SAP PSD versus** **FDT PSD (p-value)**	**<0.001**	**<0.001**	

Abbreviations: OHT = ocular hypertension; SAP = standard automated perimetry; FDT = frequency doubling technology; MD = mean deviation; PSD = pattern standard deviation, SD = standard deviation.

**Figure 1:**
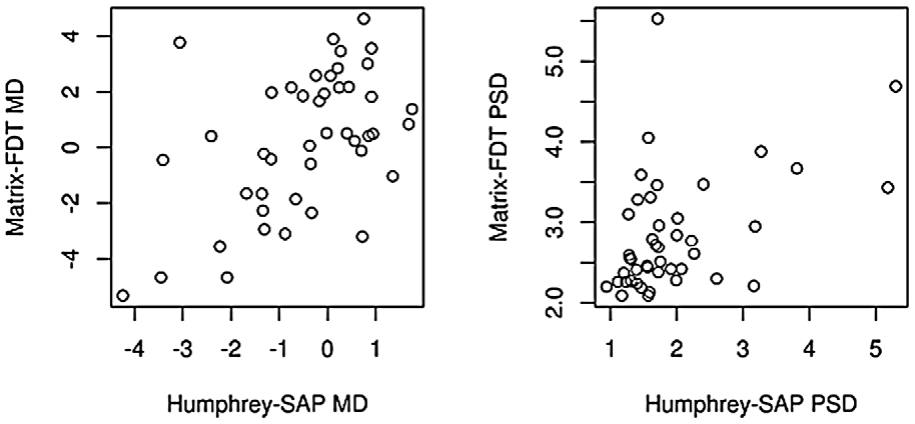
Global correlation between Humphrey-SAP and Matrix-FDT in the ocular hypertension group. Presented is the correlation between global indices (MD: mean deviation, PSD: pattern standard deviation) in OHT subjects.

Correlations between visual field global indices (mean deviation and pattern standard deviation) were analyzed using Spearman’s rank correlation coefficient (r). In the OHT group (Figure 1), average mean deviation values of Humphrey-SAP and Matrix-FDT perimeter were significantly correlated (r = 0.47, p<0.005). The average pattern standard deviation values of SAP and Matrix-FDT also showed a significant correlation (r = 0.45, p<0.005).

**Figure 2:**
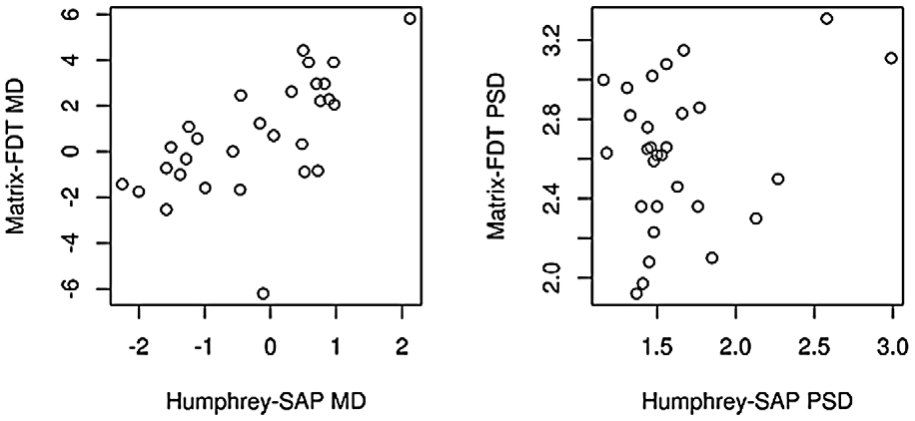
Global correlation between Humphrey-SAP and Matrix-FDT in healthy control subjects. Presented is the correlation between global indices (MD: mean deviation, PSD: pattern standard deviation) in healthy control subjects.

In the control subject group (Figure 2), only the average mean deviation values of SAP and FDT were significantly correlated (r = 0.68, p<0.001), whereas the average PSD values did not show a significant correlation (r = 0.14, p = 0.48).

Both hemifields of the control group presented with significant correlation between Humphrey-SAP and Matrix-FDT (r = 0.45, p<0.05 for superior and r = 0.36, p<0.05 for inferior hemifield). In ocular hypertensive subjects, only the inferior hemifield presented with a significant correlation (r = 0.56, p<0.001). Subdivided into quadrants, the nasal-inferior quadrants were significantly correlated in both groups (r = 0.53 for both groups, p<0.005, respectively). The temporal-inferior quadrant was significantly correlated in OHT subjects (r = 0.45, p<0.005).

**Figure 3:**
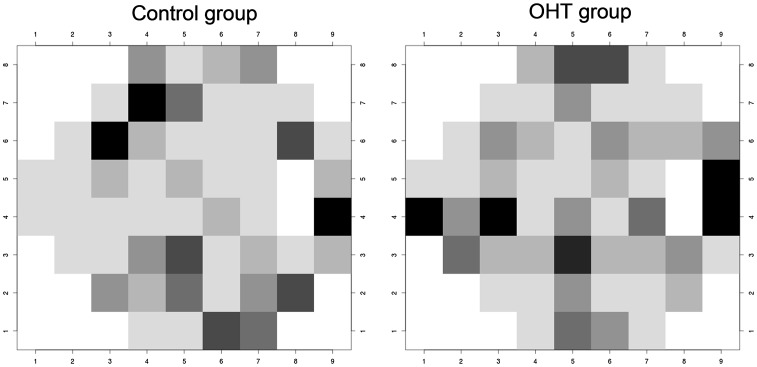
Point-wise correlation between Humphrey-SAP and Matrix-FDT in ocular hypertensive subjects (right) and healthy control subjects (left). Highly correlated visual field locations are demonstrated in black (r = 0.45 in patients and r = 0.46 in healthy subjects, p<0.01), less correlated locations are demonstrated in decreasing grey scales (r = 0.42 in both groups, p<0.02/r = 0.36 in both groups, p<0.05/r = 0.30 and r = 0.31, p<0.10/r = 0.24 in both groups, p<0.2/r = 0.16 in both groups, p<0.4).

To specifically investigate the correlation between the two instruments in the same test location of the visual field, each of the 52 SAP visual field test locations was compared with the corresponding location of the Matrix-FDT visual field as demonstrated in figure 3.

It needs to be considered that high and statistically significant correlations for given visual field points do not necessarily lead to high and statistically significant overall correlations in the given area. For instance, a greater amount of highly correlated field points can be found in the supero-nasal quadrant compared with the infero-nasal quadrant of healthy control subjects. However, a statistically significant overall correlation can only be found in the infero-nasal quadrant but not in the supero-nasal quadrant.

**Figure 4:**
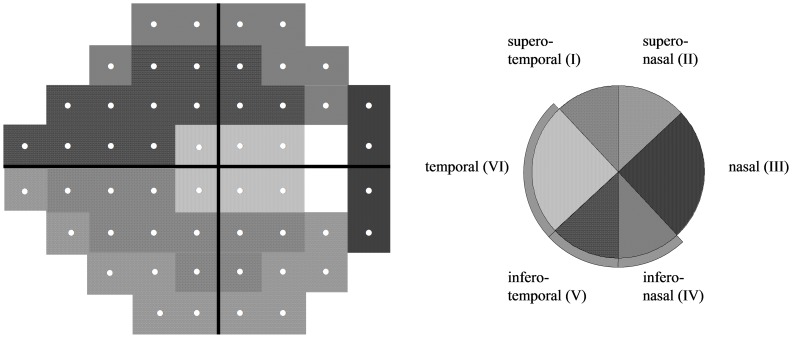
Structure-function map according to Garway-Heath et al. Visual field test points/sectors of the visual field can be related to sectors of the optic nerve head.

Garway-Heath et al. established an optic disc-visual field map which relates six sectors of the optic disc (supero-temporal, supero-nasal, nasal, infero-nasal, infero-temporal, and temporal) to corresponding sectors of the visual field (figure 4). [24] According to this map, the average of all test points belonging to one sector was evaluated in order to analyze the sectoral correlation between SAP and Matrix-FDT. Following the Garway-Heath map, it was chosen to name the sectors according to their location on the ONH, not according to their location in the visual field. For instance, the VF-temporal/ONH-nasal pair was given the name “nasal sector”. The supero-nasal disc sector corresponds to a peripheral, arcuate-shaped sector in the infero-temporal (5 field points) and the infero-nasal visual field (6 field points).

In the OHT group, SAP and Matrix-FDT correlated significantly in sectors I (supero-temporal; r = 0.49, p<0.001), II (supero-nasal; r = 0.62, p<0.001), and III (nasal; r = 0.38. p = 0.01). In the healthy subject group, there was a significant correlation between SAP and Matrix FDT in sectors I (supero-temporal; r = 0.40, p = 0.03) and V (infero-temporal; r = 0.38, p = 0.04).

## Discussion

Subjects with ocular hypertension carry an increased risk of developing glaucoma during their lifetime. It is desirable to find techniques which detect conversion from OHT to glaucoma at the earliest stage in order to avoid any significant field loss.

Matrix-FDT may detect retinal ganglion cell damage earlier than standard automated perimetry. While various investigations between SAP and FDT were carried out in glaucoma patients, only few studies addressed to OHT subjects so far.

This study investigated the correlation between SAP and Matrix-FDT in subjects with ocular hypertension and healthy control subjects. Global indices of subjects with ocular hypertension correlated significantly (MD: r = 0.47; PSD: r = 0.45; p<0.005, respectively) whereas only mean deviation values showed significant correlation in the healthy subject group (r = 0.68, p<0.001). This is in agreement with data published by Bozkurt et al. They compared ocular hypertensive participants and glaucoma patients with Humphrey 30-2 SITA standard test and Matrix 30-2 threshold test and found SAP MD positively correlated with Matrix-FDT MD (r = 0.66, p<0.001) and SAP PSD significantly correlated with Matrix-FDT PSD (r = 0.69, p<0.001). In contrast to our results, no correlation was found between SAP global indices and Matrix global indices in the OHT group. Other investigations found good correlations between SAP and Matrix-FDT in glaucoma patients. [1], [6], [9], [10] Artes et al. compared SAP and Humphrey Matrix in a small study of 15 glaucoma patients. Their global visual field indices mean deviation (MD) and pattern standard deviation (PSD) correlated highly (r = 0.86, p<0.001; r = 0.95, p<0.001, repectively). Zarkovic et al. compared both techniques in 40 patients with glaucoma and found a correlation of r = 0.69 between mean deviations. It was shown previously that there is a significant difference between the early and advanced stages of glaucoma in the degree of the correlation between SAP MD and FDT MD, with absolute MD values greater in advanced stages of glaucoma in SAP compared to FDT [25].

Concordance of glaucoma hemifield test between Matrix-FDT and SAP was 64% in OHT subjects and 66% in healthy subjects. In the OHT group, FDT presented with abnormal or borderline results in 18.2% where SAP showed normal results. In healthy subjects, this was seen in only 13.8%.

In relation to hemifields interestingly both, the superior and inferior hemifield were significantly correlated between both techniques (r = 0.45 and 0.36, p<0.05, respectively) in healthy subjects whereas in OHT subjects only the inferior hemifield showed a significant correlation (r = 0.56, p<0.001). Subdivided into quadrants, only the nasal inferior quadrant in healthy subjects (r = 0.53, p<0.005) and the temporal inferior and nasal inferior quadrants in ocular hypertensive subjects (r = 0.45 and r = 0.53, p≤0.005) were significantly correlated. This differs from the results reported by Zarkovic et al. [6] who reported the highest correlation in glaucoma patients in the superior hemifields and supero-nasal quadrants.

The correlation of SAP and FDT in individual test locations was found to be heterogenous. This is in agreement with results published by Zarkovic et al., who describe a great variance of correlation between individual test points from a correlation of less than 0.2 for some points in the inferior temporal field up to high correlated points (r = 0.88) for some points of the supero-nasal field. As seen in our investigation, they describe no apparent trend between the degree of correlation and the position of test points in relation to eccentricity from the macula, or the vertical and horizontal meridians.

Structure and function are two main pillars of glaucoma diagnosis and management. In the year 2000, Garway-Heath et al. established a structure-function map which relates sectors in the visual field to sectors on the optic nerve head. [24] The consistent use of these landmarks in both, structure and function investigations will enable a more homogenous and therefore more accurate comparison between existing and future studies on structure and function in glaucoma. Improving the magnitude of the structure-function relationship is important: Recently, the Food and Drug Administration Center for Drug Development and Research announced that it is open to using structural endpoints in clinical trials of new glaucoma drugs provided that the structural measures exhibit a strong correlation between predictability of either current visual function or future visual function [26].

According to the structure-function map by Garway-Heath et al. we found a correlation of the two instruments in the supero-temporal, supero-nasal and nasal sectors of ocular hypertensive subjects and in the supero-temporal and infero-temporal sectors of healthy control subjects. No correlation between both techniques was found in sectors IV, V, and VI of OHT subjects. These three sectors are related to the temporal, infero-temporal and infero-nasal region of the optic nerve head. In healthy subjects, sectors without correlation (II, III, IV, and VI) are arranged somewhat more heterogeneously. As early retinal nerve fiber layer defects often occur in the temporal/temporal-inferior regions of the optic nerve head [2], further investigations should elucidate whether this missing correlation in the OHT group are signs of early glaucomatous RNFL abnormalities or simply due to physiological differences in RNFL distribution in those eyes.

One factor that needs to be discussed is the role of variability in the current study. Limited agreement between techniques in relatively homogenous groups like OHT subjects and normal controls might be explained by test-retest variability. In the current study, the smallest agreement was found in temporal, infero-temporal and infero-nasal sectors of the ocular hypertensive group. This limited agreement could also be explained by an increased variability as an early sign of glaucomatous damage in these connected sectors. Interesting in this context is a study by Cellini et al. [27] They did not include SAP but compared FDT with optical coherence tomography (OCT) and pattern electroretinography (PERG) and found FDT the most sensitive and specific test for detecting early glaucomatous damage in ocular hypertensive subjects. The limited agreement in our study was found in areas where glaucoma tends to start. One could assume that Matrix-FDT was possibly more sensitive and specific than SAP for detecting early changes in these areas.

Our findings are of special interest in view of recently published data where correlations between three perimetric tests [SAP, Matrix-FDT, Flicker Defined Form Perimetry (FDF)] and confocal scanning laser ophthalmoscopy (cSLO) were investigated in glaucoma patients. [28] The highest correlation between structure (rim area, rim volume, RNFL thickness among others) and function (mean sensitivity) was found in the supero-temporal, temporal and infero-temporal regions. It is remarkable that prominent findings are again evident in the temporal and infero-temporal regions where glaucoma tends to start.

### Limitations of Our Study

Although performing several hypothesis tests using the same sample somewhat increases the experimentwise type I error in this study (which means, falsely rejecting the null hypothesis), we decided not to apply methods for correction for multiple comparisons. There is considerable controversy in the literature regarding the application of such methods. [21], [22], [23] In the presence of highly correlated variables, methods such as the Bonferroni correction are overly conservative and, although decreasing the chance of type I error, they increase the chance of type II error (that is, falsely accepting the null hypothesis).

Comparing the number of observed significant test results with the number of expected significant test results can give some impression if the findings make sense above chance level. In the current study, 80 tests were undertaken (4 tests: correlations for MD and PSD in OHT subjects and healthy control subjects for both visual field machines, 12 tests: correlations for two hemifields and four quadrants in OHT subjects and healthy control subjects for both visual field tests, 12 tests: correlations for 6 quadrants for both groups and both field machines, 52 tests: correlations between single field points for both groups and both field machines). Hence, the overall number of expected significant tests results at a significance level of 0.05 is 4. Since the number of observed significant test results is much higher than 4 (14 significant correlations for the first 28 tests and 20 significant correlations for single field points), the results can be interpreted as plausible and believable.

A weakness of this study might be the fact that only one of each field test was performed and that individuals were only familiarized with standard white-on-white perimetry. It is known that in the context of visual field testing changes in sensitivity can occur both within and between tests in normal individuals. For standard automated perimetry (SAP), variability was shown to be greater in patients with glaucoma and other optic neuropathies than in normal individuals. [17] Although there was reported a good reliability with Humphrey Matrix and with SAP perimetry in a group of glaucoma suspects with limited or no prior experience with perimetry [7], a repetition of field tests might have increased the reliability of our results. It is furthermore not possible to rule out that subjects who were assigned to the healthy subject group might show glaucomatous changes or variances which have not yet been recognized.

Finally, the sequence wherein test locations are tested might also be of importance. Locations which are tested later can either display lower sensitivity due to fatigue effects or higher sensitivity due to learning effects. Concentration, awareness and compliance of the patient as well as environmental influences can potentially bias the results.

While a large number of studies have been published in the past comparing the first-generation FDT with standard automated perimetry, only few studies have compared the second generation Matrix-FDT with SAP. The majority of these studies included glaucoma patients. To date there do not exist sufficient data that would allow a statement about visual field defects of OHT subjects in frequency doubling technology. Further studies with a higher number of participants should investigate whether the missing correlation in some parts of the optic nerve head found in this study are signs of early glaucomatous abnormalities in OHT subjects or simply due to physiological differences.

### Conclusion and Perspective

Our study found a good overall agreement between Humphrey-SAP and Matrix-FDT in ocular hypertensive and healthy control subjects. One major finding is a missing correlation between both perimeters in areas where glaucoma tends to start. As there exist only few studies comparing Matrix-FDT and SAP in ocular hypertensive subjects, our results should be confirmed by larger, prospective, longitudinal studies.

Future investigations are needed in order to compare FDT- and SAP patterns of healthy and ocular hypertensive subjects who converted to glaucoma.

## References

[1] Clement CI, Goldberg I, Healey PR, Graham S (2009) Humphrey matrix frequency doubling perimetry for detection of visual-field defects in open-angle glaucoma. Br J Ophthalmol 93: 582–588. 18669543 10.1136/bjo.2007.119909

[2] Kim TW, Zangwill LM, Bowd C, Sample PA, Shah N, et al (2007) Retinal nerve fiber layer damage as assessed by optical coherence tomography in eyes with a visual field defect detected by frequency doubling technology perimetry but not by standard automated perimetry. Ophthalmology 114: 1053–1057. 17239441 10.1016/j.ophtha.2006.09.015

[3] Harwerth RS, Carter-Dawson L, Shen F, Smith EL, Crawford ML (1999) Ganglion cell losses underlying visual field defects from experimental glaucoma. Invest Ophthalmol Vis Sci 40: 2242–2250. 10476789

[4] Johnson CA (1995) The Glenn A. Fry Award Lecture. Early losses of visual function in glaucoma. Optom Vis Sci 72: 359–370. 7566898 10.1097/00006324-199506000-00003

[5] Spry PG, Johnson CA, Mansberger SL, Cioffi GA (2005) Psychophysical investigation of ganglion cell loss in early glaucoma. J Glaucoma 14: 11–19. 15650598 10.1097/01.ijg.0000145813.46848.b8

[6] Zarkovic A, Mora J, McKelvie J, Gamble G (2007) Relationship between second-generation frequency doubling technology and standard automated perimetry in patients with glaucoma. Clin Experiment Ophthalmol 35: 808–811. 18173407 10.1111/j.1442-9071.2007.01629.x

[7] Spry PG, Hussin HM, Sparrow JM (2005) Clinical evaluation of frequency doubling technology perimetry using the Humphrey Matrix 24–2 threshold strategy. Br J Ophthalmol 89: 1031–1035. 16024860 10.1136/bjo.2004.057778 PMC1772764

[8] Johnson CA, Cioffi GA, Van Buskirk EM (1999) Frequency doubling technology perimetry using a 24–2 stimulus presentation pattern. Optom Vis Sci 76: 571–581. 10472963 10.1097/00006324-199908000-00026

[9] Bozkurt B, Yilmaz PT, Irkec M (2008) Relationship between Humphrey 30–2 SITA Standard Test, Matrix 30–2 threshold test, and Heidelberg retina tomograph in ocular hypertensive and glaucoma patients. J Glaucoma 17: 203–210. 18414106 10.1097/IJG.0b013e31815a3493

[10] Artes PH, Hutchison DM, Nicolela MT, LeBlanc RP, Chauhan BC (2005) Threshold and variability properties of matrix frequency-doubling technology and standard automated perimetry in glaucoma. Invest Ophthalmol Vis Sci 46: 2451–2457. 15980235 10.1167/iovs.05-0135

[11] Gordon MO, Beiser JA, Brandt JD, Heuer DK, Higginbotham EJ, et al.. (2002) The Ocular Hypertension Treatment Study: baseline factors that predict the onset of primary open-angle glaucoma. Arch Ophthalmol 120: 714–720; discussion 829–730. 10.1001/archopht.120.6.714 12049575

[12] Quigley HA (1998) Identification of glaucoma-related visual field abnormality with the screening protocol of frequency doubling technology. Am J Ophthalmol 125: 819–829. 9645719 10.1016/s0002-9394(98)00046-4

[13] Sample PA, Bosworth CF, Blumenthal EZ, Girkin C, Weinreb RN (2000) Visual function-specific perimetry for indirect comparison of different ganglion cell populations in glaucoma. Invest Ophthalmol Vis Sci 41: 1783–1790. 10845599

[14] Johnson CA, Samuels SJ (1997) Screening for glaucomatous visual field loss with frequency-doubling perimetry. Invest Ophthalmol Vis Sci 38: 413–425. 9040475

[15] Medeiros FA, Sample PA, Weinreb RN (2004) Frequency doubling technology perimetry abnormalities as predictors of glaucomatous visual field loss. Am J Ophthalmol 137: 863–871. 15126151 10.1016/j.ajo.2003.12.009

[16] Cello KE, Nelson-Quigg JM, Johnson CA (2000) Frequency doubling technology perimetry for detection of glaucomatous visual field loss. Am J Ophthalmol 129: 314–322. 10704546 10.1016/s0002-9394(99)00414-6

[17] Spry PG, Johnson CA (2002) Within-test variability of frequency-doubling perimetry using a 24–2 test pattern. J Glaucoma 11: 315–320. 12169968 10.1097/00061198-200208000-00007

[18] Spry PG, Johnson CA, McKendrick AM, Turpin A (2001) Variability components of standard automated perimetry and frequency-doubling technology perimetry. Invest Ophthalmol Vis Sci 42: 1404–1410. 11328758

[19] Leeprechanon N, Giangiacomo A, Fontana H, Hoffman D, Caprioli J (2007) Frequency-doubling perimetry: comparison with standard automated perimetry to detect glaucoma. Am J Ophthalmol 143: 263–271. 17178091 10.1016/j.ajo.2006.10.033

[20] Nakagawa S, Murata H, Saito H, Nakahara H, Mataki N, et al (2012) Frequency doubling technology for earlier detection of functional damage in standard automated perimetry-normal hemifield in glaucoma with low-to-normal pressure. J Glaucoma 21: 22–26. 21543995 10.1097/IJG.0b013e318202777e

[21] Nakagawa S (2004) A farewell to Bonferroni: the problems of low statistical power and publication bias. Behavioral Ecology 15 (6): 1044.1045.

[22] Bland JM, Altman DG (1995) Multiple significance tests: the Bonferroni method. BMJ 310: 170. 7833759 10.1136/bmj.310.6973.170 PMC2548561

[23] Perneger TV (1998) What’s wrong with Bonferroni adjustments. BMJ 316: 1236–1238. 9553006 10.1136/bmj.316.7139.1236 PMC1112991

[24] Garway-Heath DF, Poinoosawmy D, Fitzke FW, Hitchings RA (2000) Mapping the visual field to the optic disc in normal tension glaucoma eyes. Ophthalmology 107: 1809–1815. 11013178 10.1016/s0161-6420(00)00284-0

[25] Fukushima A, Shirakashi M, Yaoeda K, Funaki S, Funaki H, et al (2004) Relationship between indices of Humphrey perimetry and Frequency Doubling Technology Perimetry in glaucoma. J Glaucoma 13: 114–119. 15097256 10.1097/00061198-200404000-00006

[26] Weinreb RN, Kaufman PL (2011) Glaucoma research community and FDA look to the future, II: NEI/FDA Glaucoma Clinical Trial Design and Endpoints Symposium: measures of structural change and visual function. Invest Ophthalmol Vis Sci 52: 7842–7851. 21972262 10.1167/iovs.11-7895 PMC3207797

[27] Cellini M, Toschi PG, Strobbe E, Balducci N, Campos EC (2012) Frequency doubling technology, optical coherence technology and pattern electroretinogram in ocular hypertension. BMC Ophthalmol 12: 33. 22853436 10.1186/1471-2415-12-33 PMC3444883

[28] Lamparter J, Russell RA, Schulze A, Schuff AC, Pfeiffer N, et al. (2012 ) Structure-Function Relationship between FDF, FDT, SAP, and Scanning Laser Ophthalmoscopy in Glaucoma Patients. Invest Ophthalmol Vis Sci 53: 7553–7559. 23074201 10.1167/iovs.12-10892

